# Supplementary material for: Are Health Literacy and Physical Literacy Independent Concepts? A Gender-Stratified Analysis in Medical School Students from Croatia
Source: Children (Basel). 2022 Aug 15;9(8):1231. doi: 10.3390/children9081231 (PMC9406589; doi:10.3390/children9081231)
Supplement: Supplementary file 1 [file children-09-01231-s001.zip › children-1858264-supplementary.pdf]

Supplementary

Table S1. Correlations between study variables for total sample (* indicates significance of $p < 0.05$).

	1	2	3	4	5	6	7	8	9	10	11	12	13	14	15
Age (1)	1.00														
Body height (2)	-0.08														
Body mass (3)	0.03	0.53*													
BMI (4)	0.00	0.11	0.90*												
Fat mass kg (5)	-0.01	0.40*	0.95*	0.91*											
Fat mass % (6)	-0.03	0.32*	0.85*	0.84*	0.96*										
Free fat mass (7)	0.09	0.57*	0.81*	0.66*	0.65*	0.49*									
Muscle mass (8)	0.09	0.62*	0.88*	0.71*	0.69*	0.52*	0.92*								
Visceral fat (9)	-0.17	0.29*	0.86*	0.92*	0.91*	0.85*	0.52*	0.62*							
PLAYself $_{environment}$ (10)	-0.20*	0.08	-0.16*	-0.15*	-0.19*	-0.19*	-0.03	-0.09	-0.15						
PLAYself $_{self\text{-}description}$ (11)	-0.07	0.12	-0.10	-0.09	-0.14	-0.16*	-0.03	-0.04	-0.17	0.59*					
PLAYself $_{literacy}$ (12)	0.13	-0.08	0.03	0.02	0.02	0.03	0.03	0.03	0.15	0.08	0.17*				
PLAYself $_{numeracy}$ (13)	0.12	-0.10	0.00	0.01	-0.05	-0.07	0.10	0.06	-0.02	-0.04	0.13	0.33*			
PLAYself $_{physical\ literacy}$ (14)	0.03	-0.04	0.07	0.04	0.03	0.00	0.10	0.09	-0.02	0.20*	0.35*	0.32*	0.33*		
PLAYself total (15)	-0.05	0.05	-0.08	-0.08	-0.12	-0.14	0.03	-0.01	-0.12	0.67*	0.88*	0.41*	0.38*	0.58*	
HLS- EU-Q (16)	0.19*	0.03	0.08	0.12	0.10	0.11	0.08	0.04	-0.03	0.09	0.26*	0.20*	0.27*	0.12	0.31*

Legend: PLAY = Physical literacy Assessment of Youth; HLS-EU-Q = European Health Literacy Survey Questionnaire
